# *SLC28A3* expression and fludarabine-emergent autoimmune hemolytic anemia in chronic lymphocytic leukemia: A new player in an old equation

**DOI:** 10.17305/bb.2026.14208

**Published:** 2026-06-16

**Authors:** Vojin Vukovic, Teodora Karan-Djurasevic, Darko Antic, Natasa Tosic, Irena Marjanovic, Branka Zukic, Ivana Grubisa, Tamara Bibic, Kristina Tomic Vujovic, Vladimir Otasevic, Sofija Kozarac, Jelena Ivanovic, Sonja Pavlovic, Biljana Mihaljevic

**Affiliations:** 1 Clinic for Hematology, University Clinical Center of Serbia, Belgrade, Serbia; 2 School of Medicine, University of Belgrade, Belgrade, Serbia; 3 Institute of Molecular Genetics and Genetic Engineering, University of Belgrade, Belgrade, Serbia

**Keywords:** Chronic lymphocytic leukemia, autoimmune hemolytic anemia, *SLC28A3* expression, fludarabine

## Abstract

Fludarabine treatment in patients with chronic lymphocytic leukemia (CLL) has been associated with immune dysregulation and the development of autoimmune hemolytic anemia (AIHA). Prior research indicates a potential correlation between the expression of the *SLC28A3* gene, which encodes human concentrative nucleoside transporter 3 (responsible for fludarabine uptake), and treatment response. This retrospective study investigated the relationship between baseline *SLC28A3* expression levels, quantified via reverse-transcriptase polymerase chain reaction (qRT-PCR), and the incidence of AIHA in a cohort of CLL patients receiving fludarabine and cyclophosphamide (FC) therapy. Patients were categorized into two groups: “FC-emergent AIHA,” encompassing patients who developed AIHA during or following FC therapy, and “no FC-emergent AIHA,” which included patients with pre-existing AIHA prior to any treatment and those who remained AIHA-free throughout follow-up. A comparative analysis of these groups revealed no significant differences in most clinical and biological variables. However, a statistically significant difference was observed in median baseline *SLC28A3* expression, which was sevenfold lower in the “FC-emergent AIHA” group compared to the “no FC-emergent AIHA” group. Furthermore, *SLC28A3* expression did not significantly correlate with most of the other clinical or biological parameters, including time to first treatment or overall survival. These findings suggest that diminished baseline *SLC28A3* expression may correlate with an elevated risk of fludarabine-emergent AIHA in CLL patients. Further validation through larger, prospective cohorts is essential to confirm the predictive utility of *SLC28A3* expression for optimizing fludarabine-based therapeutic strategies.

## Introduction

The clinical course of chronic lymphocytic leukemia (CLL) is highly heterogeneous and can lead to various complications, including infections, autoimmune phenomena, and secondary malignancies [1]. Autoimmune hemolytic anemia (AIHA) is the most prevalent CLL-related autoimmune disorder, affecting 7%–10% of CLL patients during their disease trajectory [2]. The incidence of AIHA in CLL correlates with adverse biological indicators, such as elevated β2-microglobulin levels, unmutated immunoglobulin heavy-variable (*IGHV*) genes, and deletions of chromosome 17p (del17p) and/or 11q (del11q) [3].

The pathogenesis of CLL-related AIHA is considered multifactorial, involving humoral, cellular, and innate immunity within the specific context of altered B- and T-cell functions and their disrupted interactions [4]. In up to 90% of cases, AIHA results primarily from the production of polyclonal, high-affinity immunoglobulin G (IgG) antibodies by nonmalignant B-cell populations. These antibodies target antigens on red blood cell surfaces, leading to macrophage-mediated extravascular hemolysis. Such autoantibodies, active at 37^∘^C, are termed warm antibodies, and this form of hemolysis is known as warm AIHA (wAIHA) [5]. Conversely, CLL cells may produce monoclonal autoreactive immunoglobulin M (IgM) antibodies that bind to erythrocyte antigens at lower temperatures, triggering the complement cascade and resulting in intravascular hemolysis. This mechanism accounts for the remaining CLL-related AIHA cases, with monoclonal IgM autoantibodies referred to as cold agglutinins, and the complication termed cold AIHA (cAIHA) or cold agglutinin disease (CAD). Both AIHA types can manifest as idiopathic conditions or in association with various benign and malignant pathologies [6]. Significant alterations in the cellular immune system of CLL patients are crucial to AIHA development. Studies indicate that CLL patients exhibit increased levels of regulatory T cells (Tregs), which, along with an imbalance between Tregs and T-helper 17 (Th17) cells, may compromise immunosurveillance and foster autoimmune complications [7].

CLL-directed treatments, particularly the purine analog fludarabine, can trigger or exacerbate AIHA. It is hypothesized that fludarabine depletes Tregs, subsequently leading to a loss of suppression of autoreactive T-cell subsets. This, in turn, results in AIHA development through complex interactions with malignant and nonmalignant B cells [8]. Ysebaert et al. demonstrated severe and prolonged depletion of all T-cell subsets, with a median time for full CD4+ cell recovery extending up to 24 months post-fludarabine, cyclophosphamide, and rituximab (FCR) treatment [9]. The Leukemia Research Fund (LRF) CLL4 trial indicated that combining fludarabine with cyclophosphamide (FC) resulted in a lower incidence of AIHA (5%) compared to fludarabine or chlorambucil monotherapy (11% and 12%, respectively) [10]. In the CLL8 trial, low rates of AIHA complicating treatment were observed during or after both FC and FCR (≤ 1% of treated CLL patients) [11]. These findings suggest a protective effect of cyclophosphamide and rituximab in this context, attributable to their immunosuppressive properties [12].

The efficacy of fludarabine-based CLL treatment protocols is significantly influenced by the molecular profile of CLL cells, with del17p and/or tumor protein p53 gene (*TP53)* mutations and unmutated *IGHV* status recognized as unfavorable prognostic markers [13]. Beyond these established predictive markers, the expression of certain genes encoding nucleoside transporters, which are involved in the intracellular accumulation and metabolism of fludarabine, may also impact treatment outcomes [14, 15]. Mackey et al. revealed that high expression of the *SLC28A3* gene (solute carrier 28 A3), encoding human concentrative nucleoside transporter 3 (hCNT3), predicted a shorter time to disease progression and a lower rate of complete remission following fludarabine monotherapy [15, 16]. Furthermore, our prior study in a cohort of CLL patients without *TP53* abnormalities (del17p and/or *TP53* mutations) treated with FC demonstrated a similar effect of *SLC28A3* expression on treatment response, as patients with high *SLC28A3* expression were nearly 10 times more likely to not respond to FC than those with low expression [17].

Given the aforementioned data, we hypothesized that if *SLC28A3* gene expression influences the response to fludarabine-based therapy, it might also affect fludarabine-emergent complications, specifically AIHA. Therefore, we conducted a retrospective exploratory cohort study to assess whether baseline *SLC28A3* expression is associated with the risk of developing fludarabine-emergent AIHA in CLL patients treated with FC.

## Materials and methods

### Patients

This study included 55 CLL patients treated at the Clinic of Hematology, University Clinical Center of Serbia, between 2003 and 2018. All patients had available archived biological specimens collected from diagnosis until initiation of first-line treatment, as well as baseline clinical, laboratory, and biological data. The study adhered to the 1964 Helsinki declaration and its subsequent amendments and was approved by the Ethics Committee of the University of Belgrade School of Medicine, Belgrade, Serbia (reference number: 29/XII-6). Informed consent was obtained from all participants.

CLL diagnosis was established according to the International Workshop on Chronic Lymphocytic Leukemia (IwCLL) criteria [18]. All samples analyzed were collected at diagnosis or during the period between diagnosis and the initiation of first-line therapy. Demographic, clinical, and laboratory patient characteristics were determined at diagnosis, while molecular and cytogenetic analyses were performed either at diagnosis or prior to first treatment.

All patients received FC chemotherapy at some point in their disease course, primarily as first-line therapy. The FC regimen consisted of fludarabine 25 mg/m^2^ intravenously (IV) daily and cyclophosphamide 250 mg/m^2^ IV daily, both administered on days 1–3. Alternatively, some patients received a dose-reduced FC regimen, known as FC lite, due to age, comorbidities, or compromised renal function. This regimen comprised fludarabine 20 mg/m^2^ IV daily and cyclophosphamide 150 mg/m^2^ IV daily from days 1–3. Three patients received FC reduced to 2 days per cycle, and 4 patients received FC lite. The median number of cycles administered was 6 (range 1–6). Dosing data for 3 patients were unavailable. Response assessment was conducted according to IwCLL guidelines, with treatment outcomes defined as complete response (CR), partial response (PR), stable disease (SD), and progressive disease (PD) [18].

AIHA was diagnosed based on the following criteria: unexplained low hemoglobin level (<11 g/dL), presence of at least one indirect laboratory marker of hemolysis (high reticulocyte count, low serum haptoglobin levels, increased serum lactate dehydrogenase or bilirubin levels), and a positive direct antiglobulin test (DAT) for either IgG or C3, or the presence of cold agglutinins. For patients with a negative DAT, AIHA was diagnosed if ≥ 2 indirect signs of hemolysis were detected [19, 20]. Alternative causes of AIHA, such as active infections, other active malignancies, or concomitant autoimmune disorders, were excluded based on clinical status, relevant serological and immunological testing, clinically indicated microbiological swabs and cultures, and radiographic findings at the time of AIHA occurrence. Preexisting AIHA was defined as AIHA occurring before the initiation of first-line treatment. FC-emergent AIHA was defined as AIHA developing during or after FC treatment in patients without preexisting AIHA.

### Analytical methods

Baseline expression of the *SLC28A3* gene (i.e., expression measured in samples collected prior to any treatment initiation) was analyzed in peripheral blood mononuclear cells of CLL patients using quantitative reverse-transcriptase polymerase chain reaction (qRT-PCR) with TaqMan chemistry. Mononuclear cells were isolated from blood samples via Ficoll-Paque Plus (GE Healthcare) density-gradient centrifugation, and total cellular RNA was extracted using TRI reagent (Ambion). RNA was reverse-transcribed using RevertAid Moloney murine leukemia virus (M-MuLV) Reverse Transcriptase (Thermo Scientific) and a random hexamer primer, following the manufacturer’s instructions. qRT-PCR reactions were performed in a final volume of 10 µl with 50 ng of cDNA using TaqMan Gene Expression Assay (Applied Biosystems) for *SLC28A3* (Hs00910439_m1) and TaqMan Universal Master Mix (Applied Biosystems) in a 7500 Real-Time PCR system (Applied Biosystems). The cycling process adhered to the manufacturer’s default conditions: denaturation at 95^∘^C for 10 min, followed by 40 cycles of 95^∘^C for 15 s and 60^∘^C for 1 minute. All samples were run in duplicate. Relative *SLC28A3* mRNA expression was quantified by the comparative ddCt method, utilizing glyceraldehyde phosphate dehydrogenase (*GAPDH*) as the endogenous control gene and the HL-60 cell line as the calibrator. *SLC28A3* expression levels were reported in relative units (RU). Notably, out of the 55 patients in the study, 54 were available for expression analysis, as data could not be obtained for one sample.

The percentage of CLL cells and surface CD38 expression were determined by flow cytometry [21, 22]. Detection of cytogenetic abnormalities characteristic of CLL (del13q14, trisomy 12, del11q22-23, and del17p13) was performed by fluorescence *in situ* hybridization (FISH) on interphase nuclei obtained from peripheral blood using a panel of locus-specific probes (Vysis/Abbott Laboratories) according to the manufacturer’s instructions. The somatic hypermutational (SHM) status of clonotypic *IGHV* genes, B-cell receptor (BCR) stereotypy, and *TP53* mutational status were analyzed as recommended by the European Research Initiative on CLL (ERIC) [23–25].

### Ethical statement

The research was conducted with the approval of the institutional medical ethics committee (No. 29/XII-6) and in accordance with the 1964 Helsinki Declaration and its subsequent amendments.

### Statistical analysis

The prespecified primary analysis aimed to examine the association between the development of FC-emergent AIHA and baseline expression of *SLC28A3*. Secondary analyses investigated the association of FC-emergent AIHA and *SLC28A3* expression with clinical and biological variables in CLL patients identified at diagnosis or prior to the first line of therapy.

Categorical variables, presented as absolute numbers and frequencies, were analyzed using Fisher’s exact test. Continuous variables, presented as medians with ranges and 95% confidence intervals (95% CI), were analyzed using Student’s *t*-test, Mann–Whitney rank-sum test, and one-way analysis of variance (ANOVA) on ranks. The Shapiro–Wilk test assessed data distribution. Relationships between continuous variables were analyzed using the Spearman rank-order correlation test.

Time to first treatment (TTFT) was defined as the period from diagnosis to the initiation of first-line therapy. Overall survival (OS) was measured from diagnosis to death from any cause or last follow-up. Survival analyses were performed using the Kaplan–Meier method, the log-rank test, and Cox regression analysis.

Receiver operating characteristic (ROC) analysis evaluated the discriminatory power of *SLC28A3* expression as a predictor of FC-emergent AIHA development. Internal validation of the ROC analysis was performed using the percentile bootstrap method with 2000 stratified bootstrap replicates.

All statistical analyses were conducted using SPSS 21.0 software (IBM), except for bootstrap validation of ROC analysis, which was performed in R using the pROC package. For all analyses, *P*-values were two-tailed, and significance was defined as *P* < 0.05.

## Results

### Characteristics of the cohort

This study included 55 CLL patients treated with FC chemotherapy: 39 men and 16 women (male-to-female ratio = 2.44) aged 38–75 years at diagnosis (median = 57 years). The clinical and biological characteristics of the patients are summarized in Table 1.

**Table 1:** Clinicobiological features of CLL patients with and without FC-emergent AIHA

**Variable**	**All patients** **(*n* ═ 55)**	**No FC-emergent AIHA** **(*n* ═ 48)**	**FC-emergent AIHA** **(*n* ═ 7)**	***P*-value**
Sex (*n* ═ 55)^a^, male/female, no.	39/16	35/13	4/3	0.402^e^
Age (years), (*n* ═ 55)^a^, median (range)	57 (38-75)	57 (38-75)	53 (42-62)	0.198^d^
WBC [x10^9^/l ], (*n* ═ 53)^a^, median (range)	47.1 (7.1-570)	45.9 (7.1-570)	64.6 (16.5-324.3 )	0.763^c^
Ly [x10^9^/l ], (*n* ═ 48)^a^, median (range)	38.9 (2.7-558.6)	37.3 (2.7-558.6)	59 (8.4-285.4)	0.483^c^
Platelets [x10^9^/l ], (*n* ═ 50)^a^, median (range)	174.5 (1-432)	165 (1-432)	192 (76-263)	0.617^d^
Hb [g/l], (*n* ═ 50)^a^, median (range)	128 (27-163)	129 (27-161)	124.5 (117-163)	0.591^c^
ESR (*n* ═ 38)^a^, median (range)	17 (2-150)	16 (2-150)	22 (4-32)	0.905^c^
CRP (*n* ═ 27)^a^, median (range)	3.3 (0.1-36.5)	3.2 (0.1-36.5)	4.1 (2.5-8.2)	0.241^c^
β2-M [mg/l] (*n* ═ 39)^a^, median (range)	4 (0.2-11.5)	4 (0.2-11.5)	2.9 (2-4.9)	0.124^c^
LDH [U/L], (*n* ═ 47)^a^, median (range)	383 (81-1042)	429 (207-1042)	353 (81-398)	0.088^c^
Binet stage (*n* ═ 54)^a^, no. (%)				0.390^e^
A	18 (33.3)	14 (29.8)	4 (57.1)	
B	25 (46.3)	23 (48.9)	2 (28.6)	
C	11 (20.4)	10 (21.3)	1 (14.3)	
CD38 status (*n* ═ 50)^a^ **,** no. (%)				0.387^e^
positive (≥30 %)	19 (38)	18 (40.9)	1 (16.7)	
negative (< 30 %)	31 (62)	26 (59.1)	5 (83.3)	
Cytogenetic risk (*n* ═ 55)^a^, no. (%)				0.722^e^
favorable (del13q14^b^ )	9 (16.4)	7 (14.6)	2 (28.6)	
Intermediate (no aberrations, trisomy 12)	33 (60)	29 (60.4)	4 (57.1)	
unfavorable (del11q22–23, del17p13)	13 (23.6)	12 (25)	1 (14.3)	
*TP53* mutations (*n* ═ 55)^a^, no. (%)	8 (14.5)	7 (14.6)	1 (14.3)	1.000^e^
*IGHV* SHM status (*n* ═ 54)^a^, no. (%)				0.322^e^
mutated	11 (20.4)	11 (23.4)	0 (0)	
unmutated	43 (79.6)	36 (76.6)	7 (100)	
BCR stereotypy (*n* ═ 53)^a^, no. (%)	9 (17)	7 (15.2)	2 (28.6)	0.588^e^
*SLC28A3* expression ^10^, (*n* ═ 54)^a^, median (range)	0.034 (0.001-0.54 0)	0.042 (0.001-0.54)	0.006 (0.006-0.021)	**0.002^c^

^a^The number in brackets represents the total patient count for whom parameter values at diagnosis were accessible. ^b^Deletion 13q14, detected by FISH, occurring as the sole chromosomal abnormality. ^c^Mann-Whitney *U*-test. ^d^Student’s *t*-test. ^e^Fisher’s exact test. **Abbreviations:** AIHA, autoimmune hemolytic anemia; BCR, B-cell receptor; β2-M, beta-2 microglobulin; CRP, C-reactive protein; del11q22–23, deletion of chromosome 11q22–23; del13q14, deletion of chromosome 13q14; del17p13, deletion of chromosome 17p13; ESR, erythrocyte sedimentation rate; FC, fludarabine plus cyclophosphamide; Hb, hemoglobin; *IGHV*, immunoglobulin heavy-chain variable region; LDH, lactate dehydrogenase; Ly, lymphocyte count; SHM, somatic hypermutation; *SLC28A3*, solute carrier family 28 member 3; *TP53*, tumor protein p53 gene; WBC, white blood cell count.

During a median follow-up of 72 months (range = 4–142 months), 38 patients (69.1%) died, 14 patients (25.5%) were alive, and 3 patients (5.4%) were lost to follow-up. The median OS was 76 months (95% CI = 68.1–83.9).

The majority of patients (45/55; 81.8%) received FC as first-line treatment, 6 patients (10.9%) as second-line, and 4 patients (7.3%) as third- or fourth-line treatment. TTFTs ranged from 0–71 months, with a median of 6 months (95% CI = 0.9–11.1). The overall response rate to the first therapeutic line was 78.2%; CR, PR, SD, and PD were observed in 41.8%, 36.4%, 12.7%, and 9.1% of patients, respectively.

AIHA was documented in 13 (23.6%) patients in our cohort. For 6 patients (46.2% of patients with hemolysis; 10.9% of the total cohort), AIHA occurred before any treatment (preexisting AIHA). In contrast, 7 patients (53.8% of patients with hemolysis; 12.7% of the total cohort) developed FC-emergent AIHA. Among these 7 patients, hemolysis occurred during FC therapy in 5 (2 patients during first-line therapy and 3 patients during later-line therapy), while in the remaining 2 patients, hemolysis developed 17 and 28 months after first-line FC treatment initiation (during first remission). Notably, in all patients with preexisting AIHA, hemolysis resolved before the start of first-line treatment, and none subsequently developed FC-emergent AIHA.

Considering all documented AIHA cases (7 males, 6 females; median age at diagnosis = 59 years), no associations were observed between this complication and any clinical or laboratory parameters at diagnosis (sex, age, blood cell counts, hemoglobin, β2-microglobulin, serum lactate dehydrogenase, erythrocyte sedimentation rate (ESR), C-reactive protein). However, a trend toward higher median lymphocyte counts at diagnosis was detected in patients who developed AIHA compared to those without AIHA (*P* ═ 0.074, Mann–Whitney rank-sum test). Among molecular and cellular prognostic factors (CD38, chromosomal abnormalities, *IGHV* SHM status, BCR stereotypy, *TP53* mutations) and *SLC28A3* expression, AIHA was significantly associated only with unmutated *IGHV* status (*P* ═ 0.048, Fisher’s exact test). A trend toward shorter OS was also observed in patients with AIHA compared to those without (54 vs. 80 months, respectively; *P* ═ 0.086, log-rank test).

### FC-emergent AIHA is associated with lower *SLC28A3* expression

In our cohort, FC-emergent AIHA occurred in 7 patients (4 males, 3 females; median age at diagnosis = 54 years), all of whom had the *IGHV*-unmutated CLL subtype.

To test the hypothesis that FC-emergent AIHA is associated with *SLC28A3* expression, we first investigated whether baseline *SLC28A3* levels differed among patients with preexisting AIHA, those with FC-emergent AIHA, and those who did not experience AIHA during follow-up. This analysis revealed significant differences in *SLC28A3* levels among the three groups (*p* ═ 0.006, one-way ANOVA on ranks) (Figure 1). Pairwise comparisons showed no significant difference in *SLC28A3* expression between patients with preexisting AIHA and those without AIHA (*P* ═ 0.556, Mann–Whitney rank-sum test). However, significant differences were observed when comparing patients with preexisting AIHA to patients with FC-emergent AIHA (*P* ═ 0.007, Student’s *t*-test), and when comparing patients without AIHA to patients with FC-emergent AIHA (*P* ═ 0.002, Mann–Whitney rank-sum test).

**Figure 1.**
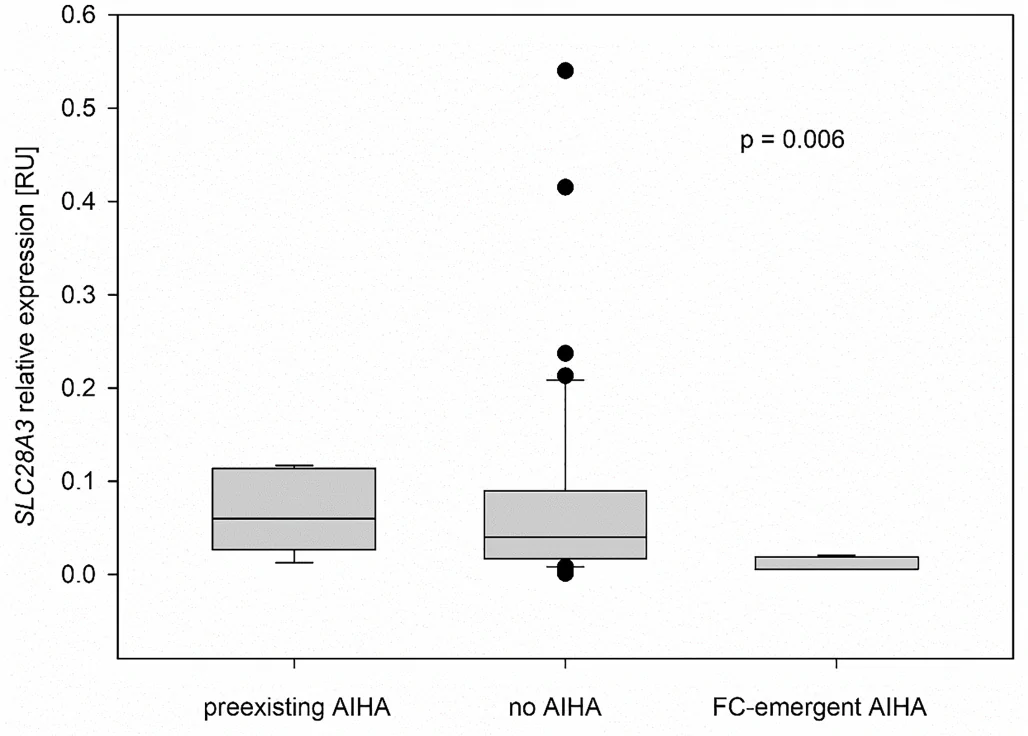
**Baseline *SLC28A3* expression according to AIHA status in FC-treated CLL patients.** Baseline *SLC28A3* mRNA expression was quantified by qRT-PCR in peripheral blood mononuclear cells collected before initiation of any treatment from 54 CLL patients and expressed as RU. Patients were classified into three groups: Preexisting AIHA, defined as AIHA documented before first-line therapy (*n* ═ 6); no AIHA, defined as absence of AIHA during follow-up (*n* ═ 41); and FC-emergent AIHA, defined as AIHA developing during or after FC treatment in patients without preexisting AIHA (*n* ═ 7). Boxes represent interquartile ranges, horizontal lines within boxes represent medians, whiskers indicate non-outlier ranges, and dots indicate outlying values. *SLC28A3* expression differed significantly among the three groups (*P* ═ 0.006, one-way ANOVA on ranks), with the lowest expression observed in patients with FC-emergent AIHA. Median expression values (range) were 0.060 (0.013–0.117) RU in the preexisting AIHA group, 0.040 (0.001–0.540) RU in the no-AIHA group, and 0.006 (0.006–0.021) RU in the FC-emergent AIHA group. **Abbreviations:** AIHA, autoimmune hemolytic anemia; ANOVA, analysis of variance; CLL, chronic lymphocytic leukemia; FC, fludarabine plus cyclophosphamide; mRNA, messenger RNA; qRT-PCR, quantitative reverse-transcription polymerase chain reaction; RU, relative units; *SLC28A3*, solute carrier family 28 member 3.

**Figure 2.**
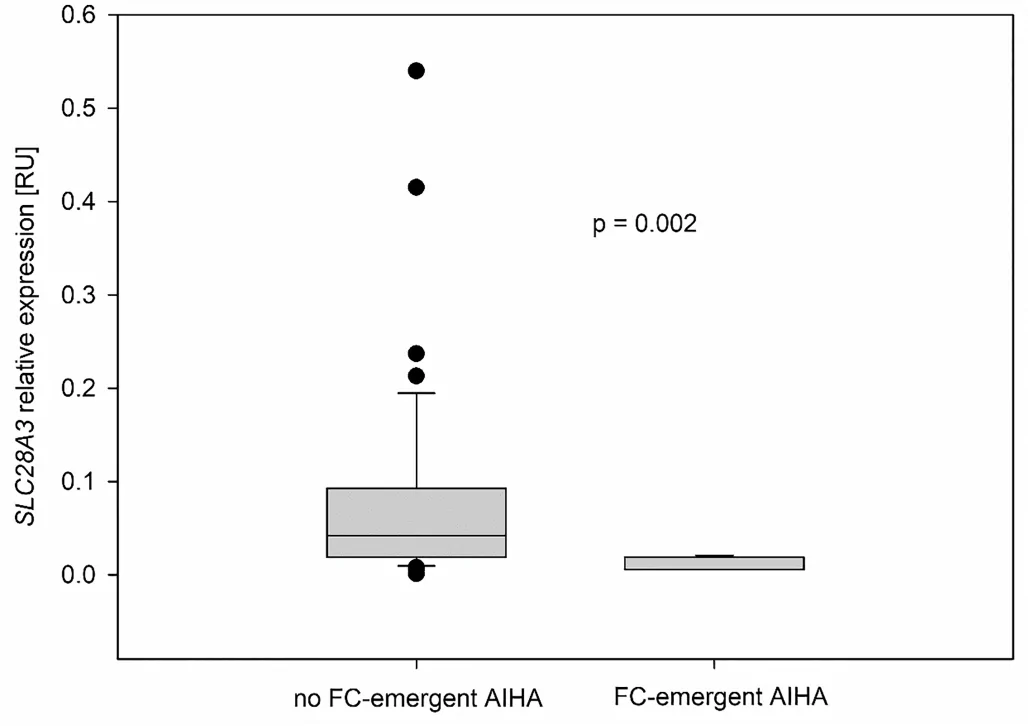
**Baseline *SLC28A3* expression is lower in CLL patients who develop FC-emergent AIHA.** Baseline *SLC28A3* mRNA expression was quantified by qRT-PCR in peripheral blood mononuclear cells collected before initiation of any treatment from 54 CLL patients treated with FC and expressed as RU. The no FC-emergent AIHA group included patients who did not develop AIHA during follow-up and patients with preexisting AIHA, defined as AIHA occurring before first-line therapy and not recurring during or after FC treatment. FC-emergent AIHA was defined as AIHA developing during or after FC treatment in patients without preexisting AIHA. Boxes represent interquartile ranges, horizontal lines within boxes represent medians, whiskers indicate non-outlier ranges, and dots indicate outlying values. Patients with FC-emergent AIHA had significantly lower baseline *SLC28A3* expression than those without FC-emergent AIHA (*p* ═ 0.002, Mann–Whitney rank-sum test). Median expression values (range) were 0.042 (0.001–0.540) RU in the no FC-emergent AIHA group (*n* ═ 47) and 0.006 (0.006–0.021) RU in the FC-emergent AIHA group (*n* ═ 7). **Abbreviations:** AIHA, autoimmune hemolytic anemia; CLL, chronic lymphocytic leukemia; FC, fludarabine plus cyclophosphamide; mRNA, messenger RNA; qRT-PCR, quantitative reverse-transcription polymerase chain reaction; RU, relative units; *SLC28A3*, solute carrier family 28 member 3.

Given that hemolysis did not occur during or after FC treatment in patients with preexisting AIHA, we further divided our cohort into two groups: the “no FC-emergent AIHA” group (comprising patients who did not experience AIHA and those with preexisting AIHA) and the “FC-emergent AIHA” group (comprising patients in whom AIHA emerged during or after FC administration). A comparison of these two groups revealed similar clinical and biological variables (Table 1). The only detected difference was in *SLC28A3* mRNA expression, which was significantly lower in the “FC-emergent AIHA” group than in the “no FC-emergent AIHA” group (*P* ═ 0.002, Mann–Whitney rank-sum test) (Figure 2). Specifically, the median *SLC28A3* mRNA expression level in the “FC-emergent AIHA” group was 7-fold lower than in the “no FC-emergent AIHA” group. Importantly, this difference in *SLC28A3* expression remained significant even when the two cases where AIHA developed after first-line FC treatment (during first remission) were excluded from the analysis (*P* ═ 0.015, Mann–Whitney rank-sum test). ROC analysis indicated that baseline *SLC28A3* expression was associated with FC-emergent AIHA status in this cohort, yielding an area under the curve (AUC) of 0.8708 (95% CI = 0.7661–0.9755) with a corresponding sensitivity of 1 (95% CI = 0.5904–1) and specificity of 0.72 (95% CI = 0.5736–0.8438) at the optimal cut-off (Figure 3). Following the bootstrapping procedure, the resulting 95% CIs were 0.7538–0.962 for AUC, 0.4286–1 for sensitivity, and 0.5957–0.8723 for specificity, demonstrating relatively stable discriminatory performance within the study cohort. Using the ROC-derived cut-off value of 0.0215 RU, patients were categorized as low- or high-*SLC28A3* expressers to examine the distribution of FC-emergent AIHA cases across expression categories. This approach revealed a predominance of high vs low expressers in the “no FC-emergent AIHA” group (34 and 13 patients, respectively), whereas all 7 patients in the “FC-emergent AIHA” group expressed low levels of *SLC28A3* mRNA (*P* < 0.001, Fisher’s exact test) (Figure 4).

**Figure 3.**
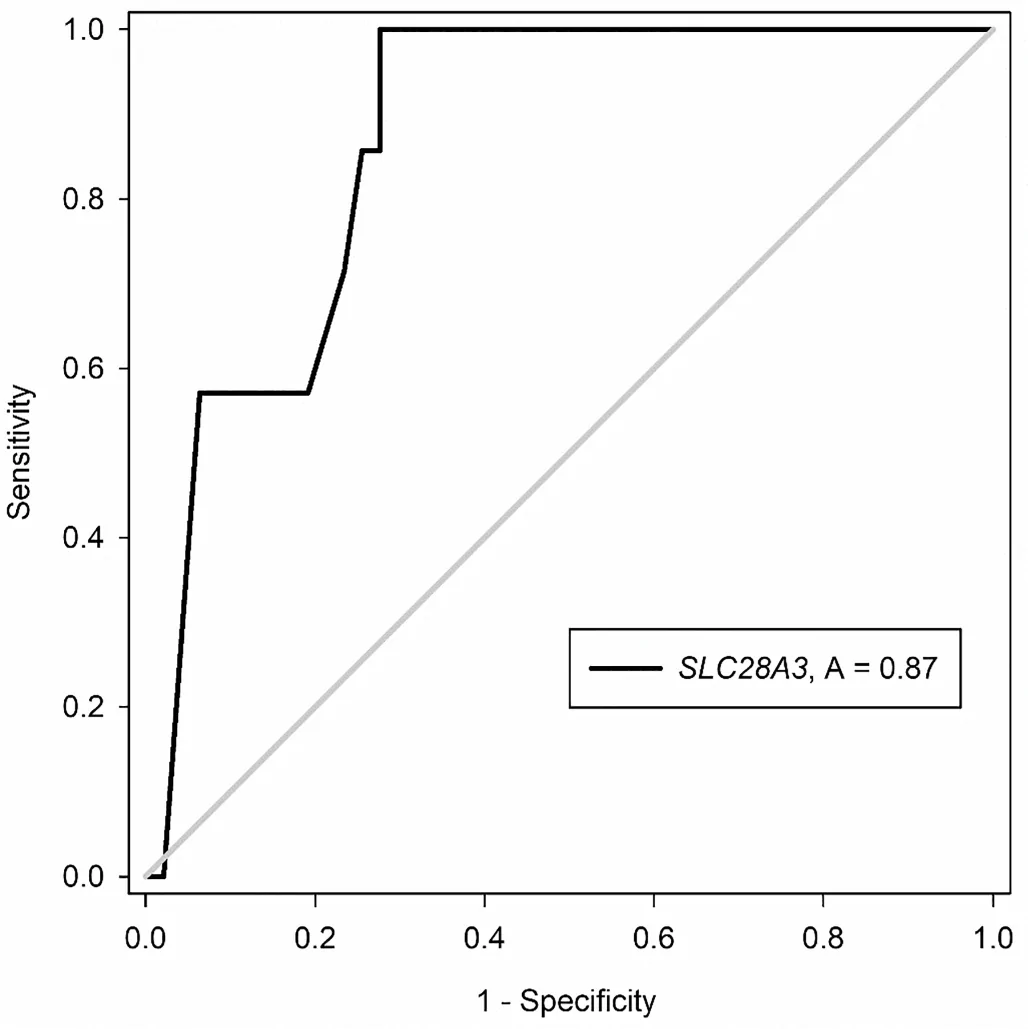
**ROC analysis of baseline *SLC28A3* expression for FC-emergent AIHA in FC-treated CLL patients.** Baseline *SLC28A3* mRNA expression was quantified by qRT-PCR in peripheral blood mononuclear cells collected before initiation of any treatment from 54 patients with CLL treated with fludarabine plus cyclophosphamide. ROC analysis was used to assess the discriminatory performance of *SLC28A3* expression for FC-emergent AIHA status, defined as AIHA developing during or after FC treatment in patients without preexisting AIHA. The black curve represents the ROC curve, and the gray diagonal line represents no discrimination. Baseline *SLC28A3* expression showed good discriminatory performance for distinguishing patients with FC-emergent AIHA (*n* ═ 7) from those without FC-emergent AIHA (*n* ═ 47): AUC = 0.8708, 95% CI = 0.7661–0.9755; *p* ═ 0.001679. The optimal ROC-derived cut-off was 0.0215 RU, with sensitivity of 1.000, 95% CI = 0.5904–1.000, and specificity of 0.7234, 95% CI = 0.5736–0.8438. Because this was an exploratory retrospective analysis, the cut-off should be considered hypothesis-generating rather than clinically validated. **Abbreviations:** AIHA, autoimmune hemolytic anemia; AUC, area under the curve; CI, confidence interval; CLL, chronic lymphocytic leukemia; FC, fludarabine plus cyclophosphamide; mRNA, messenger RNA; qRT-PCR, quantitative reverse-transcription polymerase chain reaction; ROC, receiver operating characteristic; RU, relative units; *SLC28A3*, solute carrier family 28 member 3.

**Figure 4.**
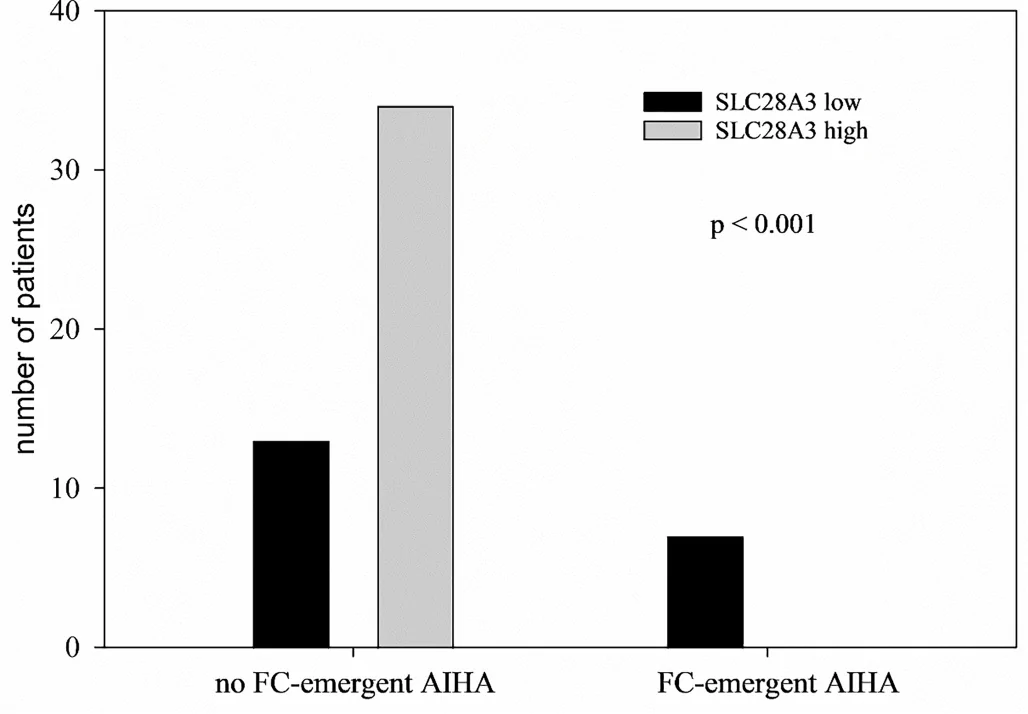
**Distribution of *SLC28A3* expression categories according to FC-emergent AIHA status in CLL patients.** Baseline *SLC28A3* mRNA expression was quantified by qRT-PCR in peripheral blood mononuclear cells collected before initiation of any treatment from 54 CLL patients treated with fludarabine plus cyclophosphamide and expressed as RU. Patients were stratified as *SLC28A3*-low or *SLC28A3*-high using the exploratory ROC-derived cut-off of 0.0215 RU. The no FC-emergent AIHA group included patients who did not develop AIHA during follow-up and patients with preexisting AIHA that did not recur during or after FC treatment. FC-emergent AIHA was defined as AIHA developing during or after FC treatment in patients without preexisting AIHA. Bars represent the number of patients in each *SLC28A3* expression category. Low *SLC28A3* expression was significantly enriched in the FC-emergent AIHA group: All 7 patients with FC-emergent AIHA were *SLC28A3*-low, compared with 13 of 47 patients in the no FC-emergent AIHA group; 34 patients in the no FC-emergent AIHA group were *SLC28A3*-high, whereas no patient with FC-emergent AIHA showed high *SLC28A3* expression (*P* < 0.001, Fisher’s exact test). Because the cut-off was derived from this retrospective exploratory cohort, it should be interpreted as hypothesis-generating rather than clinically validated. **Abbreviations:** AIHA, autoimmune hemolytic anemia; CLL, chronic lymphocytic leukemia; FC, fludarabine plus cyclophosphamide; mRNA, messenger RNA; qRT-PCR, quantitative reverse-transcription polymerase chain reaction; ROC, receiver operating characteristic; RU, relative units; *SLC28A3*, solute carrier family 28 member 3.

Since the ROC-derived cut-off was not clinically established and was used solely for exploratory stratification, *SLC28A3* expression was treated as a continuous variable in all subsequent analyses. Notably, baseline *SLC28A3* expression levels were not associated with the majority of analyzed baseline clinical and biological prognostic factors, nor with TTFT and OS, when the entire cohort was considered. However, a negative correlation between *SLC28A3* expression and lymphocyte count at diagnosis (r ═ –0.359, *P* ═ 0.0133, Spearman rank-order correlation test) was observed. Additionally, lower *SLC28A3* levels were detected in patients expressing stereotyped BCRs compared to those expressing heterologous BCRs (*P* ═ 0.017, Mann–Whitney rank-sum test). Our cohort included 9 patients (16.4%) with stereotyped BCRs, all of whom had the *IGHV*-unmutated CLL subtype, belonging to subsets #1 (4 patients), #5 (2 patients), #3, #31, and #99 (1 patient each). Interestingly, the sole patient with subset #3 BCR expressed a low level of *SLC28A3* and developed FC-emergent AIHA after second-line FC therapy. We also attempted regression-based analyses to investigate potential confounding factors affecting the association between *SLC28A3* expression and FC-emergent AIHA. However, the small number of FC-emergent AIHA events resulted in unstable estimates with wide confidence intervals, indicative of quasi-complete separation, precluding reliable interpretation. Consequently, these analyses are not presented.

## Discussion

Fludarabine-induced AIHA was well-documented in CLL patients treated with single-agent fludarabine in the late 1990s [26, 27]. However, the integration of cyclophosphamide and rituximab into fludarabine-based chemoimmunotherapy appears to mitigate this potential for inducing hemolysis. Large randomized trials have reported an AIHA incidence of less than 1% in patients receiving FC or FCR [11]. Nevertheless, a study from the MD Anderson Cancer Center observed an AIHA incidence of 5.8% during or after FCR in CLL patients, underscoring the ongoing clinical relevance of this issue [12].

The last decade has witnessed a paradigm shift in CLL treatment, with most patients now receiving targeted agents such as Bruton tyrosine kinase inhibitors and Bcl2 inhibitors (e.g., venetoclax) [28, 29]. Despite this, Thompson et al. recently demonstrated that a subset of fit patients with favorable molecular features continues to achieve impressive long-term responses and benefits from FCR [30]. Nonetheless, due to compelling results from randomized prospective trials confirming the significant advantage of targeted therapies (alone or in combination with monoclonal antibodies) over chemoimmunotherapy, the latter is no longer broadly recommended for CLL treatment. Its use is primarily restricted to regions where targeted therapies (e.g., Bruton tyrosine kinase [BTK] and B-cell lymphoma 2 [BCL2] inhibitors) are not fully accessible [28, 31–35]. Given that many countries worldwide still face reimbursement restrictions that limit access to these agents to varying degrees based on regional and economic factors, FCR remains a relevant treatment option for a substantial proportion of CLL patients globally. This continued reliance on FCR highlights the enduring importance of understanding and managing fludarabine-emergent adverse events, including AIHA, in routine clinical practice.

Fludarabine is administered as a 5’ monophosphate derivative, which is dephosphorylated by 5’-nucleotidase to F-ara-A before cellular entry via specific human nucleoside transporters (hNTs) [14]. Among the seven known nucleoside transporters (NTs), four human equilibrative NTs (hENTs 1–4) and three human concentrative NTs (hCNTs 1–3) have been identified as crucial for active fludarabine transport across the cell plasma membrane, specifically hENT1, hENT2, and hCNT3 [36]. Mackey et al. investigated the relationship between the relative expression of seven genes encoding proteins involved in fludarabine metabolism (hENT1, hENT2, hCNT3, deoxycytidine kinase, and three 5’-nucleotidases: ecto-5’-nucleotidase, deoxynucleotidase-1, and cytoplasmic high-K_m_5-nucleotidase) and treatment outcome in 45 patients receiving fludarabine monotherapy [15]. Of these genes, only the relative expression level of the gene encoding hCNT3 (*SLC28A3*) exhibited a significant yet inverse association with treatment response and time to disease progression (TDP). Specifically, higher relative levels of *SLC28A3* mRNA correlated with significantly lower rates of complete response and shorter TDP following fludarabine treatment [15]. Our group corroborated these findings, revealing a similar association between higher baseline *SLC28A3* expression and treatment failure in a cohort of 44 *TP53* aberration-free CLL patients treated with FC [17]. Although initial hypotheses posited that increased NT expression would enhance treatment outcomes by concentrating active fludarabine metabolites within leukemic cells, both Mackey et al. and our group obtained seemingly paradoxical results. Furthermore, Mackey et al. demonstrated that hCNT3, found to be expressed only in the cytoplasm of a limited percentage of CLL patients, lacks nucleoside transport activity in the plasma membrane of CLL cells [15]. This finding complicated the interpretation of their results. The authors speculated that the strong association between hCNT3 gene expression and fludarabine resistance might stem from mutual regulation of *SLC28A3* with other genes implicated in fludarabine resistance. Another hypothesis, supported by the confirmed cytoplasmic abundance of hCNT3, suggests that hCNT3 may localize to intracellular organelle membranes, such as mitochondria, thereby modulating fludarabine toxicity. An alternative explanation proposes that increased production and/or migration of hCNT3 to the plasma membrane, induced by heightened fludarabine exposure, provides a “salvage” nucleoside transport mechanism, minimizing the effects of intracellular nucleoside depletion caused by fludarabine [15].

In this study, we analyzed *SLC28A3* expression in CLL patients who developed AIHA during or after FC treatment (“FC-emergent AIHA”) and compared them to patients who developed AIHA before first-line treatment or never experienced AIHA (“no FC-emergent AIHA”). Two patients who developed AIHA 17 and 28 months post-FC, respectively, were included in the “FC-emergent AIHA” group. This inclusion was based on consistent data regarding deep and long-term CD4+ cell depletion following FCR treatment and the absence of documented CLL progression in both patients at the time of AIHA onset [9, 37]. Our results revealed a 7-fold lower baseline *SLC28A3* expression in patients with FC-emergent AIHA compared to the “no FC-emergent AIHA” group. It is crucial to emphasize that *SLC28A3* expression was assessed only once at baseline, either at diagnosis or prior to the initiation of first-line therapy. Consequently, for patients who received FC in later treatment lines, the analyzed *SLC28A3* levels reflect expression at disease onset rather than immediately preceding the FC course associated with AIHA development. Given the unavailability of longitudinal samples and the current lack of data on temporal changes in *SLC28A3* expression during CLL progression or under treatment pressure, our findings should be interpreted as suggesting an association between baseline *SLC28A3* expression and subsequent FC-associated AIHA. They do not directly demonstrate that *SLC28A3* expression at the time of FC exposure predicts AIHA risk. Nevertheless, the observed association may imply that baseline *SLC28A3* expression captures an underlying biological susceptibility that remains clinically relevant throughout disease evolution. This possibility warrants confirmation in prospective studies incorporating serial sampling.

It should be noted that several potential confounding factors may contribute to the development of FC-emergent AIHA, including advanced disease stage, older age, prior autoimmune phenomena, adverse cytogenetic abnormalities (del17p and del11q), unmutated *IGHV* status, high absolute lymphocyte count, elevated β2-microglobulin levels, and increased CD38 expression [38]. Other potential risk factors for AIHA in CLL patients include gender and prior treatment exposure. However, retrospective cohort studies have yielded conflicting results regarding the association between gender and AIHA risk [39, 40]. Concerning prior treatment exposure, Visentin et al. reported a higher frequency of autoimmune disorders, including AIHA, among previously treated CLL patients, although without specifying the treatment modality or line of therapy [39]. This association is plausible, as previously treated patients are more likely to harbor adverse disease biology than treatment-naïve individuals, to have a longer disease duration, and consequently to exhibit more profound immune dysregulation, all of which may contribute to an increased risk of AIHA [6]. Unfortunately, the relatively small sample size in our study limited our ability to assess potential confounders in a multivariable model. Furthermore, analyses based on AIHA severity and the presence of Evans syndrome were not feasible due to missing data. Consequently, the potential influence of these factors on the observed association between *SLC28A3* expression and FC-emergent AIHA could not be assessed and should be addressed in larger studies with more comprehensive clinical data. Given that *SLC28A3* expression also influences treatment outcome, it could be speculated that the same mechanism(s) underlying the association of lower *SLC28A3* expression with a better response to fludarabine-based therapy may also be involved in fludarabine toxicity, particularly the development of FC-emergent AIHA, in *SLC28A3* low-expressing patients. However, this possibility requires further exploration, as some studies have shown that improved effectiveness of chemo(immuno)therapy reduced the frequency of AIHA [41].

Additionally, it is important to emphasize that the pathophysiology of AIHA in CLL is not solely determined by the impact of CLL cells on red blood cells but rather by a complex process of immune dysregulation caused by the disease, in which both polyclonal B cells and different T-cell subsets play crucial roles [6]. These non-leukemic lymphocytes, particularly T cells, are integral to the specific CLL microenvironment and contribute to the development of various complications, such as infections and autoimmune phenomena [42]. Historically, fludarabine, in addition to affecting CLL B cells, has been shown to decrease the levels of both CD4+ and CD8+ T lymphocytes in treated patients [43]. Moreover, it leads to an imbalance between Th17 and T regulatory cells, thereby contributing to the pathophysiology of fludarabine-emergent AIHA [6]. Currently, fludarabine is widely employed as a lymphodepleting agent in patients undergoing chimeric antigen receptor T (CAR-T) therapy [44]. To further elucidate the mechanisms of fludarabine-emergent AIHA in the context of *SLC28A3* expression, it would be particularly valuable to analyze hCNT3 expression and activity in different subsets of non-leukemic lymphocytes, both of T and B phenotypes. However, these investigations were not performed in this study or, to the best of our knowledge, elsewhere. Furthermore, recently published studies have highlighted the significance of clonal hematopoiesis (CH) in CLL, demonstrating that myeloid-lineage CH is associated with disease course, treatment efficacy, and treatment-related complications in CLL patients. It has been confirmed that, unlike targeted agents, chemoimmunotherapy promotes CH in the myeloid compartment, which may translate into a higher risk of secondary myeloid malignancies. Clonal hematopoiesis in CLL was also shown to influence the T cell population by reducing T cell exhaustion and promoting a proinflammatory T cell transcriptional signature [45, 46]. In the context of the previously discussed pathogenesis of AIHA, it may be hypothesized that the presence of CH in CLL patients contributes to immune dysregulation that may facilitate the development of AIHA, particularly following chemoimmunotherapy. Thus, correlating *SLC28A3*expression with CH in CLL patients with AIHA may be a valuable avenue for future research.

Our results further support the established association between unmutated *IGHV* status and AIHA in CLL patients [47–49]. Given that unmutated *IGHV* status is linked to more aggressive disease, it was not surprising that patients with AIHA in our cohort experienced shorter OS compared to those who did not develop AIHA, consistent with previous studies [10, 47, 50, 51]. Additionally, our patient assigned to stereotyped subset #3 exhibited low levels of *SLC28A3* and developed FC-emergent AIHA. This finding aligns with the observations of Maura et al., who reported an increased risk of AIHA development in CLL patients expressing subset #3 BCR [49].

## Conclusion

In conclusion, our study identified a low baseline *SLC28A3* expression level as a potential biomarker to identify CLL patients at risk of FC-emergent AIHA, thereby potentially helping to prevent this harmful complication. Although its applicability might be limited to a subset of CLL patients with favorable molecular profiles who remain candidates for fludarabine-based treatment, it could prove particularly useful in developing regions where novel targeted therapies are less accessible or affordable. However, our findings should be interpreted cautiously due to the retrospective design and small sample size. Accordingly, this study should be considered exploratory and hypothesis-generating rather than definitive. Further studies on larger, prospective cohorts are warranted to confirm our findings and to elucidate the underlying mechanisms linking nucleoside transporter expression to fludarabine-associated autoimmune hemolytic anemia.

## Data Availability

The data are available from the corresponding author upon request.
